# Assembly of the Genome of the Disease Vector *Aedes aegypti* onto a Genetic Linkage Map Allows Mapping of Genes Affecting Disease Transmission

**DOI:** 10.1371/journal.pntd.0002652

**Published:** 2014-01-30

**Authors:** Punita Juneja, Jewelna Osei-Poku, Yung S. Ho, Cristina V. Ariani, William J. Palmer, Arnab Pain, Francis M. Jiggins

**Affiliations:** 1 Department of Genetics, University of Cambridge, Cambridge, United Kingdom; 2 Computational Bioscience Research Center, KAUST, Thuwal, Kingdom of Saudi Arabia; National Institute of Allergy and Infectious Diseases, United States of America

## Abstract

The mosquito *Aedes aegypti* transmits some of the most important human arboviruses, including dengue, yellow fever and chikungunya viruses. It has a large genome containing many repetitive sequences, which has resulted in the genome being poorly assembled — there are 4,758 scaffolds, few of which have been assigned to a chromosome. To allow the mapping of genes affecting disease transmission, we have improved the genome assembly by scoring a large number of SNPs in recombinant progeny from a cross between two strains of *Ae. aegypti*, and used these to generate a genetic map. This revealed a high rate of misassemblies in the current genome, where, for example, sequences from different chromosomes were found on the same scaffold. Once these were corrected, we were able to assign 60% of the genome sequence to chromosomes and approximately order the scaffolds along the chromosome. We found that there are very large regions of suppressed recombination around the centromeres, which can extend to as much as 47% of the chromosome. To illustrate the utility of this new genome assembly, we mapped a gene that makes *Ae. aegypti* resistant to the human parasite *Brugia malayi*, and generated a list of candidate genes that could be affecting the trait.

## Author Summary

Mosquitoes are important for transmission of human diseases including dengue and yellow fever. The sequencing of the genomes of key mosquito species including *Aedes aegypti* has helped us to understand the factors that allow mosquitoes to vector disease. While the genome for *Ae. aegypti* has been sequenced, it is in many pieces which have not yet been arranged on chromosomes. To this end, we have created a genetic linkage map and measured the distance between genetic markers, which allows us to assign them to regions of the genome. Using this method, we also detected errors in the current genome sequences. We used our genetic map to find regions of the mosquito genome associated with the development of *Brugia malayi*, a nematode that causes lymphatic filariasis in humans. A better genome assembly will be important for the development of novel methods for controlling disease transmission.

## Introduction

The mosquito *Aedes aegypti* is a vector of the most important arboviral pathogens that infect humans, including dengue, yellow fever and chikungunya viruses [1]. Dengue is estimated to occur in 50 million people each year, representing a 30-fold increase over the past 50 years [1], [2]. Originally from Western Africa, this highly invasive vector species was spread by trade and travel throughout Africa from the 15^th^ century, Asia from the 18^th^ century, and worldwide in the past 70 years [3], [4]. *Ae. aegypti* was subject to a continental eradication effort from 1948 to 1962 which led to its virtual elimination from 21 countries in the Americas [5]–[7]. However, today the mosquito has become re-established, with dengue once again found in most of this range. Alternatives to conventional control efforts include widespread releases of transgenic males that lead to sterility in their offspring with wild females [8], and of mosquitoes infected with the bacterium *Wolbachia*, which increases the resistance of the mosquito to infection [9], [10]. The development of these and subsequent control techniques would be benefited by improvement of the available genomic resources.

To be able to identify the genetic components of vector competence, knowledge of the genome organization of the mosquito host is essential. Among the first three sequenced genomes of mosquito vectors, *Anopheles gambiae* is the most complete and organized genome draft. The *Ae. aegypti* and *Culex quinquefasciatus* genomes are still highly fragmented, with large numbers of scaffolds (supercontigs) not yet ordered and oriented across chromosomes [11]–[13]. At 1.38 Gigabases, the *Ae. aegypti* genome is the largest among these sequenced mosquito genomes, and the genome is currently organized into 4,758 scaffolds. There is a long history of genetic and physical mapping in *Ae. aegypti* [14]–[19], and using these data approximately 31% of the genome was preliminarily assigned to chromosomes using physical and genetic markers at the time of publication of the genome assembly [11], [14]. More recently, improved physical mapping techniques combined with existing genetic linkage maps were used to place 100 scaffolds representing 13% of the genome with confidence to specific regions within chromosomes [20]. The difficulty in assembling the *Aedes* and *Culex* genomes is attributable to their large chromosomes and the presence of a high percentage of repetitive transposable elements [11], [12]. With many of the scaffolds not having been assigned to chromosomes, it confounds attempts to map genes of interest.

*Ae. aegypti*, as a model system for studying mosquito-parasite interactions, shows variation in susceptibility to *Brugia* parasites, one of the genera of filarial nematodes that causes lymphatic filariasis [21]–[23]. Of particular epidemiological interest is the discovery that the genetic control of susceptibility to *Brugia* is similar to the more pan-tropic filarial parasite *Wuchereria bancrofti* [21]–[23], which causes about 90% of human lymphatic filariasis cases. This genetic susceptibility of *Ae. aegypti* to *Brugia* infections has been shown to follow a Mendelian mode of inheritance, with a major effect sex-linked resistance locus [15], [21] that maps onto the first chromosome of *Ae. aegypti* [17]. In *Ae. aegypti*, sex is determined by an allele on the homomorphic first chromosome, with a genotype of Mm in males and mm in females. The locus determining resistance to *B. malayi* has been defined to a 10 cM region, which is estimated to cover ≈17 Mb of the genome [14], [17], and is approximately 4 cM away from the sex determination locus [15], . Although resistance to *B. malayi* has been mapped, the lack of chromosomal scaffolds limits the identification of polymorphism underlying these traits. Mapping and isolation of the gene could potentially provide a useful tool for comparative analyses of vector populations and encourage a better understanding of vector competence in different mosquito populations.

The advent of improved DNA-based technology for genetic and linkage mapping [16], [24] has enhanced the identification of loci that affect the vector competence of mosquitoes. For example, an integrated genetic map based on microsatellite analyses identified three loci as responsible for melanotic encapsulation of *Plasmodium cynomolgi* in *An. gambiae* [25], [26]. In *Ae. aegypti*, susceptibility to *Brugia malayi* was found to be associated with two quantitative trait loci (QTL) using Restriction Fragment Length Polymorphism (RFLP) [17]. We contribute to efforts to improve the genetic map of *Ae. aegypti* by using Restricted-site Associated DNA (RAD) sequencing [27]. We have assigned numerous scaffolds to chromosomes and ordered the scaffolds along on the chromosomes. We also identified errors in the current genome assembly, such as misassemblies of contigs into scaffolds and misassignments of scaffolds to chromosomes, and we illustrate the utility of this new genome assembly by re-mapping of the genetic susceptibility of *Ae. aegypti* to *B. malayi*.

## Materials and Methods

### Mosquito strains

We used several different strains of *Ae. aegypti* to generate a genetic linkage map and to map *B. malayi* resistance onto this map. The Liverpool IB12 (LVP-IB12) strain of *Ae. aegypti* is a highly inbred line that was used for the genome sequencing project and was initiated from a line that was susceptible to infection by *B. malayi* [11]. It is derived from the Liverpool strain which has been maintained in culture since 1936 and was originally collected from West Africa [21]. The COSTA RICA strain is a wild-type strain originally collected from Puntarenas, Costa Rica, in 2001 [28]. Both strains were obtained from the Malaria Research and Reference Reagent Resource Center (MR4) (ATCC, Manassas, Virginia, USA). COSTA RICA was inbred by single pair mating for two generations to reduce heterozygosity (CR-IB2).

LVP-IB12 was found to be resistant to infection by *B. malayi*, suggesting that resistance was segregating in the founding line and became fixed during inbreeding. We confirmed the resistance phenotype by testing a separate isolate obtained from David Severson whose laboratory originally performed the inbreeding. A *B. malayi* susceptible strain of Liverpool (subsequently referred to as LVP-FR3) was obtained from the NIAID/NIH Filariasis Research Reagent Resource Center (FR3, Atlanta, Georgia, USA). This strain is used by the FR3 to maintain *B. malayi* in culture.

### Crossing design for linkage map construction

We used a backcrossing design between LVP-IB12 and CR-IB2 to generate a mapping population for producing a genetic linkage map. An individual virgin female LVP-IB12 was mated to an individual male CR-IB2 (P*_0_*). Female F_1_ individuals (n = 38) were then backcrossed to male LVP-IB12 (n = 38) by mass mating. Female progeny (n = 99) of this backcross were used to generate a linkage map. P_0_ parents and backcrossed individuals were frozen at −80°C until DNA extraction could be performed.

### Crossing design and infections for pooled QTL mapping of Brugia resistance

We used a separate cross between LVP-IB12 (resistant) and LVP-FR3 (susceptible) for QTL mapping, taking advantage of the fact that both lines originated from the same strain and are expected to be genetically similar. An individual LVP-IB12 male was mated to five LVP-FR3 females (P_0_), and eggs were collected separately for each P_0_ mother. Susceptibility is known to be recessive and sex-linked [15], [17], [21], so F_1_ females were backcrossed to susceptible males to ensure that the trait was segregating in the mapping population. F_1_ females from the same mother were backcrossed in groups of five to individual LVP-FR3 males, and eggs were collected en masse from these groups of five F_1_ mothers. P_0_ and F_1_ parents were frozen at −80°C until DNA extraction could be performed.

For infections, *B. malayi* was obtained from Darren Cook and Mark Taylor at the Liverpool School of Tropical Medicine (LSTM), and donated human blood was obtained from Blood Transfusion Services at Addenbrooke's Hospital, Cambridge, UK. Backcrossed mosquitoes and control mosquitoes from each of the parental stocks were fed on blood containing parasites at a concentration of 125 microfilariae per 20 µl of blood. Females were 5–7 days old on the day of infection. Unfed mosquitoes were discarded, and infected mosquitoes were maintained on a 10% fructose solution for 11 days post-infection. To check for infection status, individual mosquitoes were separated at the head and thorax and incubated in 100 µl of 1× PBS for one hour at 37°C. At this point the supernatant was transferred to a microscope slide and the number of L3 parasites was counted, and the mosquito carcasses were stored at −80°C until DNA extraction could be performed.

After DNA extraction, pools of DNA were created that contained equal amounts of 9–13 refractory or susceptible backcrossed individuals for a total of 27 refractory pools and 14 susceptible pools. Individuals were always pooled within families.

### RAD library construction

DNA was extracted using QiaAmp MicroDNA kit (Qiagen) with the following modifications. Tissues were incubated with RNAse post-homogenization and no carrier RNA was used. DNA was eluted in 50 µl AE buffer and 1 µl of eluate was quantified with Qubit 2.0 fluorimeter (Invitrogen). For the P_0_ male only, DNA was whole genome amplified (WGA) using V2 Genomiphi kit (GE Healthcare, UK) in three parallel reactions, yielding a total of 3.7 µg of DNA.

We adapted the RAD-tag protocol developed by [27], making minor changes for use with *Ae. aegypti*. We used PstI, which has ∼257 k predicted cut sites in the *Ae. aegypti* genome, for constructing our linkage map, and SbfI, which has ∼14 k predicted cut sites, for pooled QTL mapping. Each restriction digestion reaction tube contained 5 µl of Buffer 4 (NEB), 0.2 µl PstI-HF, X µl DNA (0.17–1 µg) and deionized water to make 50 µl total reaction volume. 7.9 µl of P1 adaptor was ligated onto the DNA fragments with T4 ligase (NEB). For the linkage map construction, P1 barcoded backcrossed individuals with similar DNA concentrations were pooled into groups of 9 prior to continuing. For the WGA P_0_ male, a single library was prepared using 19 different P1 adaptors. For QTL mapping, pools of refractory or susceptible individuals were given unique P1 adaptors and a single library was prepared for each family. An additional library was prepared using individually P1 barcoded P_0_ and F_1_ parents. P1 ligated DNA was sheared for 6.5 minutes at high intensity with a Bioruptor Sonication System (Diagenode) and loaded on agarose gels. An insert size between 300–500 bp was excised and purified using the QIAquick MinElute Gel Purification kit. DNA was blunt-end repaired (Quick Blunting Buffer, NEB) and A-tailed (Taq, NEB) before P2 adaptor ligation. 4–12 µl of DNA library was amplified in a 100 µl reaction volume as described in [27].

For linkage map construction, backcrossed progeny were sequenced in 13 lanes of 100 bp paired-end HiSeq2000 (KAUST). In some cases, PhiX was added to increase sequence diversity. The P_0_ male library was sequenced in a single lane of 100 bp paired-end HiSeq2000 (KAUST). For QTL mapping, libraries were pooled and sequenced in a single lane of 100 bp paired-end HiSeq2000 (EASIH, Addenbrooke's, Cambridge, UK).

### Alignment of RAD data for linkage map construction

Individuals or pools of individuals were sorted using the FastX Barcode Splitter [29] while allowing up to 1 mismatch to the barcode sequence, and sequence originating from the barcode was clipped. Sequences were quality trimmed from the 3′ end using Trimmomatic version 0.20 [30] when average quality scores in sliding windows of 4 base pairs dropped below 15. Sequences less than 36 base pairs in length were discarded.

We obtained 2.6 billion reads for 98 individuals in the mapping population, and 168 million reads for the CR-IB2 parental male. Sequences were aligned to the reference genome (AaegL1, Oct 2005) [11] with BWA version 0.6.1-r104 [31] using the default parameters. Alignments for individuals sequenced across different lanes were merged into single BAM files using Picard version 1.66. Alignments were sorted, indexed, and assigned read groups using samtools version 0.1.18 [32] and Picard. Indels were realigned using GATK version 1.5 [33], and PCR and optical duplicates were removed using Picard. Libraries had high rates of PCR and optical duplication, with duplicates accounting for 22% to 90% of the sequences. After duplicate removal, a total of 944 million reads remained for the mapping population, ranging from 13,880 to 29.7 million reads per individual.

### SNP calling and marker selection for linkage map construction

GATK's CallableLoci was used to identify callable regions of the CR-IB2 male parent. Callable regions were defined as having 20 or more reads with mapping qualities higher than 23 and fewer than 4 improperly paired reads. SNPs were simultaneously called in these regions in the CR-IB2 male parent and 98 mapping progeny individuals using GATK's UnifiedGenotyper with the default parameters. VCFtools version 0.1.7 [34] was used to discard sites with a SNP quality less than or equal to 20 or with more than two alleles present. Genotype calls in individuals with a quality score greater than 20 and a minimum depth of 9 were retained, yielding a total of 277,735 segregating sites.

SNP markers were retained on the basis of parental genotype and mapping progeny segregation patterns. We assumed that the LVP-IB12 female parent was homozygous for the reference allele (denoted LVP/LVP) and retained 92,457 SNPs where the CR-IB2 male parent was homozygous for the non-reference allele (denoted CR/CR). In this backcrossed design, it is expected that progeny will never be CR/CR and that the ratio of LVP/LVP homozygotes to LVP/CR heterozygotes would be approximately 0.5. In practice, a large number of sites had one or more CR/CR progeny, which likely due to residual heterozygosity leading to inheritance of the CR allele through the LVP-IB12 stock. We applied strict filters to limit markers to those of the highest quality. We discarded markers with fewer than 20 genotyped progeny, where any progeny was CR/CR homozygous, and where the ratio of LVP/LVP homozygotes to LVP/CR heterozygotes was less than 0.3 or greater than 0.7. After these filtering steps, 9,761 sites remained.

Even with high average coverage (>10× in 67 individuals, >20× in 46 of our individuals), there was unevenness of coverage between loci in individuals leading to large amounts of missing data. To maximize the number of markers, we inferred P_0_ parent of origin for tags, contigs or scaffolds using SNP calls present in individuals. We collapsed multiple SNPs present in a tag originating from a single restriction enzyme cut site to a single marker, discarding the genotype if there was a conflict within a tag. This resulted in 5,222 unique tags. We further collapsed multiple markers in a contig to a single marker, again discarding the genotype if there was a conflict within a contig. Lastly, we collapsed multiple markers in a scaffold to a single genotype if all markers were in agreement (retaining the original number of markers for building the map), and we retained the original contig markers if there was a conflict. In this way, if a single marker was genotyped at any position in a tag, contig, or scaffold, then the entire region could be assigned to a parent of origin. After this, 2,826 high quality markers were left on 749 scaffolds.

### Linkage map construction

MSTMap was used to construct our linkage map as it has been shown to perform robustly in the presence of genotyping errors and missing data [35]. MSTMap was run with the population type set to DH, the distance function set to Kosambi, the objective function set to COUNT, and the minimum p-value set to 0.00001. Isolated linkage groups were created when up to 15 markers were found to be separated by more 3 cM from the rest of the map. We enabled the detection of bad data and disabled estimation before clustering. Map building worked best with less missing data, so the 35 individuals with the least missing data (<500 missing genotypes) were selected and a maximum of two individuals were allowed to have missing genotypes for each marker. Markers with up to four missing genotypes were used to assign scaffolds to chromosomes without assigning a position.

MSTMap always yielded three major linkage groups and sometimes also produced isolated linkage groups of bad markers. Map building was run iteratively, each time removing these genotypes and isolated linkage groups detected by the program until they were all removed. For the final maps, we visually inspected the map and removed double recombination events that were supported by 3 or fewer markers as these always occurred with isolated clusters of homozygous genotypes and were most likely caused by the relatively low coverage cutoff of 9×. We also found regions of the map with an excess of heterozygotes which upon further inspection were found to contain cases where the CR allele was inherited via the LVP parent (determined by considering the multi-SNP inheritance pattern at the locus), leading to a heterozygote call for what was truly a homozygous region. We removed these markers and those with identical segregation patterns. The final map from MSTMap consisted of 2,006 markers.

### Misassemblies

**Figure 1:**
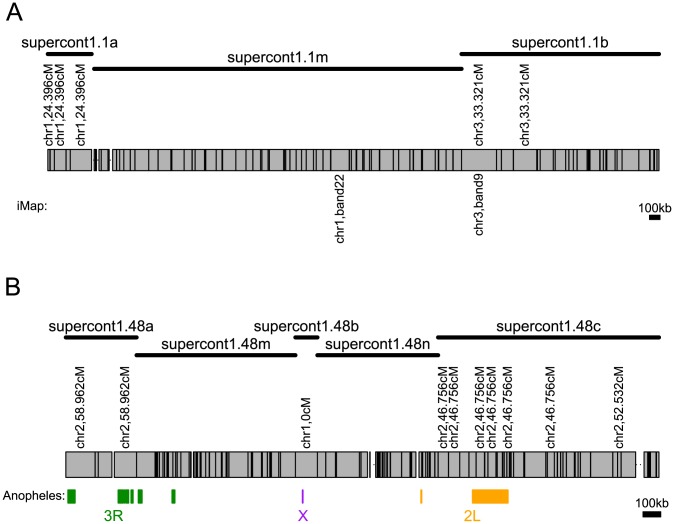
Examples of two misassembled scaffolds. Individual contigs are shown as gray rectangles. Contigs with markers in this study are indicated with an * above the scaffold and are labeled with their position on the genetic map. The new scaffolds created by splitting misassemblies (identified in this study only) are shown with solid lines. New scaffolds with suffixes ‘a,’ ‘b,’or ‘c’ contain markers that allow placement on the genetic map. New scaffolds with suffixes ‘m’ or ‘n’ fall between conflicting markers and therefore contain a misassembly and cannot be placed on the genetic map. A) Supercontig 1.1 with markers from the previously published integrated map [20] shown below. The mapping of markers to two different chromosomes indicates a misassembly within the scaffold, which is supported by both markers sets. B) Supercontig 1.48 with synteny with *An. gambiae* shown below the scaffold and the different colors indicating different chromosome arms. Both our markers and syntenic breaks with *An. gambiae* indicate that this scaffold is misassembled in at least two instances.

Where misassemblies were detected in the existing *Ae. aegypti* genome, we removed them from the genome assembly by splitting the scaffold. Preliminary analysis found no evidence for misassemblies within contigs, so scaffolds were split at the gaps between contigs. We only detected one internal misassembly, where a contig mapping to one location was flanked by contigs mapping to an alternate location. Therefore, in all cases, we split the scaffolds at the misassembly junctions, turning one scaffold into two or more pieces (Figure 1). If markers did not occur on consecutive contigs, then the exact position of the misassembly could not be determined and the region between the markers was renamed with an “m,” “n,” etc to denote misassembly. The remaining parts of the scaffold with no additional evidence for a misassembly were renamed “a,” “b,” etc (eg supercont1.1 became supercontig1.1a, supercontig 1.1b, and supercontig 1.1m, see Figure 1). To account for these misassemblies in subsequent experiments, we generated a new reference fasta which was used for later alignments (available upon request).

Linkage groups were assigned to chromosomes based on comparison with the most recent available map for *Ae. aegypti* [20] (Table S1). In cases where a scaffold was misassembled, the scaffold fragment containing the marker position in [20] was used for the comparison.

### Pooled QTL mapping

Reads were aligned as described earlier, except this time mapping to the modified reference that accounted for scaffold misassemblies. PCR duplication rates ranged from 44–94%. Reference and alternate allele counts were extracted from the VCF file produced by GATK. We selected the 12 resistant pools and 9 susceptible pools with the highest levels of coverage, and only retained sites with 10 or more reads per pool. This yielded a total of 6,868 sites. Significance was tested in R using a general linear model with phenotype as a fixed effect and the proportion sequence reads with the SNP allele found in the genome as the response variables. The error structure was set to quasibinomial. A *P*-value cutoff was determined by permuting the phenotype of the DNA pools (infected or uninfected) 10,000 times and recalculating *P*-values, retaining the minimum value across all the markers from each permutation. Based on this, the cutoff for a genome-wide significance of P<0.01 was set at an individual SNP significance of *P*<1.7×10^−6^.

### Synteny and recombination rates

Synteny to *Anopheles gambiae* was determined by comparing the genetic position of 1∶1 orthologous genes (>70% identity) in *Ae. aegypti* with the physical position in *An. gambiae*. Gene annotations and orthologies were obtained from VectorBase [36].

Recombination rates were estimated as the local slope of a curve fit to plots of genetic versus physical distances for each chromosome. Estimates were obtained by using the loess function to locally adjust polynomial curves and implemented by the MareyMap package in R [37].

## Results and Discussion

### Genetic map

**Figure 2:**
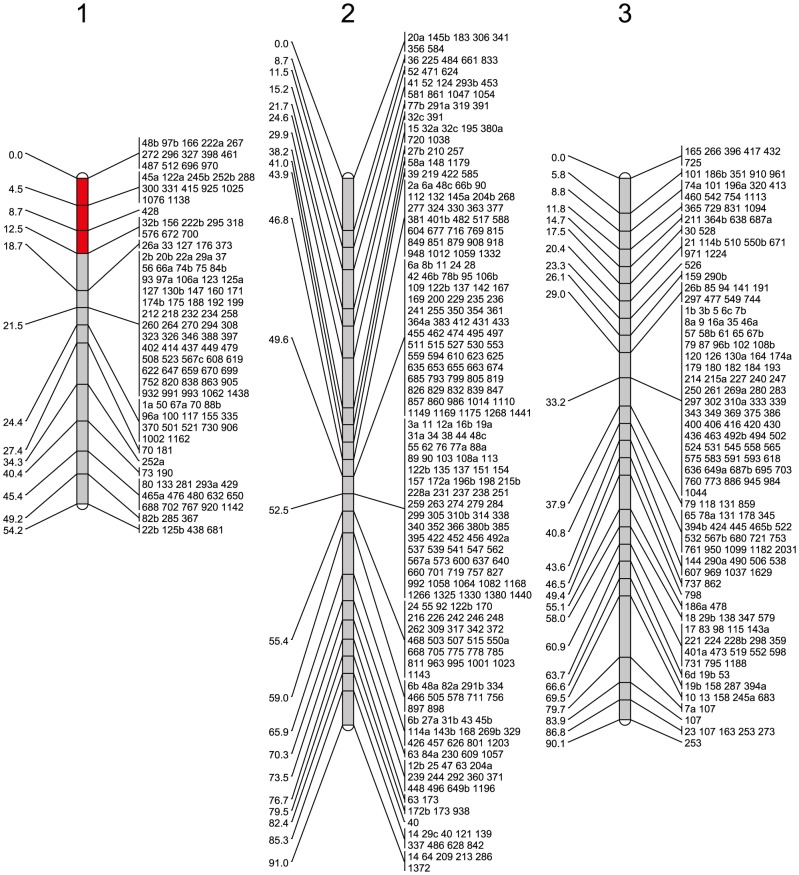
A linkage map of *Ae. aegypti*. The map was constructed using SNPs identified in RAD tag sequences. Positions in cM are indicated to the left of each linkage group. Scaffolds mapping to each position on the map are shown to the right of each linkage group and are named by the last 2–4 digits of their supercontig ID number. The region linked with resistance to infection by *B. malayi* is highlighted in red. Scaffolds with suffixes a–d were previously misassembled and have been split and assigned new scaffold names (see Figure 1 and Table S2 for additional information).

We reconstructed a genetic map of *Ae. aegypti* using over 2,000 markers, which were scored by sequencing RAD tags and filtered using a series of steps to ensure that only the most reliable genotype calls were retained. The genetic map consisted of three linkage groups, which represent the three chromosomes of *Ae. aegypti* (Figure 2, Table S2). The total length of the genetic map is 235 cM (Table 1), which is similar to that reported in previous studies [14].

**Table 1:** Summary of our assembly of the genome onto a genetic map.

	Chr 1	Chr 2	Chr 3	Total
**Chromosome + position on linkage map** 1 **:**				
scaffolds^2^	147	309	202	658
Mb	177	364	265	806 Mb (58%)
genes	2,096	4,768	3,560	10,424 (60%)
**Chromosome only** 3 **:**				
scaffolds^2^	163	339	225	727
Mb	187	383	290	912 Mb (66%)
genes	2,232	4,983	3,903	11,118 (64%)
cM	54.2	91	90.1	235 cM
cM/Mb	0.306	0.249	0.291	0.291

1 scaffolds which have been ordered along the chromosome (see Figure 2, Table S2).

2 number of mapped scaffolds after splitting misassemblies.

3 scaffolds assigned to the chromosome only (see Table S2).

### Misassemblies in the genome

The large number of SNPs used in linkage map construction allowed us to detect misassemblies in the published *Ae. aegypti* genome by looking for cases where a single scaffold contains contigs that map to different regions of the genome. To be classified as a misassembly, we required markers within a single scaffold either to map to different linkage groups or to regions >10 cM apart on the same linkage group. We find that 14% (63 of 435) of scaffolds with more than one marker are misassembled within the current genome assembly (AaegL1). These errors in the genome were corrected by splitting the scaffold where it was misassembled (Figure 1; see Methods for details).

These results suggest that a notable percentage of the genome is misassembled, including 6 of the 10 largest scaffolds in the assembly. Misassembled scaffolds are on average larger in size than non-misassembled scaffolds, and this effect is independent of the number of markers on the scaffold (Figure S1). This is expected since long scaffolds have more opportunities to be misassembled. Of the misassemblies we detect, 43 are misassemblies where contigs within a scaffold map to two different chromosomes, 18 map to different regions within the same chromosome, and 3 map to three different places in the genome. We also find that in 23 of 63 (37%) of the misassembled scaffolds, the break junction is within 3 contigs from the beginning or end of the scaffold (Table S3). This could be because the beginnings and ends of scaffolds are be supported by fewer links.

The finding that a large number of scaffolds are misassembled is consistent with the previous study by [20], which found misassemblies in 3 of 7 scaffolds with more than one marker. Our data confirm one of these misassemblies, splitting supercont1.1 between chromosomes 1 and 3 as was previously shown (Figure 1A). Given the 4,000 scaffolds with fewer than two markers, it is expected that we have not detected all of the misassemblies in the genome. However, larger scaffolds tend to have more markers, and these larger scaffolds represent a sizeable fraction of the genome (Figures S1, S2).

### Assembling the genome onto the genetic map

**Figure 3:**
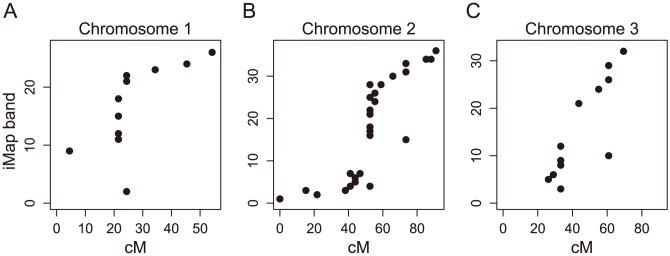
The correlation between the cM position of a scaffold on our genetic map and its position on the previously published integrated map [20]. The positions of scaffolds on chromosomes 1 (A), 2 (B), and 3 (C) are significantly correlated between the map presented here and the previously published map order (Spearman correlation, A: ρ = 0.803, *P* = 0.0017; B: ρ = 0.899, *P* = 0<0.0001; C: ρ = 0.857, *P* = 0.0004). Outliers are potentially caused by unidentified scaffold misassemblies. In cases where we found a scaffold to be misassembled, the correlation was performed by using the genetic map position of the contig closest to the one used in [20]. In one instance on Chromosome 2 and two instances on Chromosome 3, scaffolds mapped to a different chromosome in [20] than on our genetic map and these are not included in this analysis.

Having split misassembled scaffolds, we used SNPs to assign the scaffolds to chromosomes and order the scaffolds along the chromosome. In total we have placed 589 scaffolds onto the genetic map (Figure 2, Tables 1, S2), including 531 scaffolds that were not included on the most recently published map. The map order is highly consistent with a recently published map that integrated the genetic, physical, and genomic maps of the previous decades [20] (Figure 3; Spearman correlation, Chromosome 1: ρ = 0.803, *P* = 0.0017; Chromosome 2: ρ = 0.899, *P* = 0<0.0001; Chromosome 3: ρ = 0.857, *P* = 0.0004). Because of low rates of recombination, the orientations of individual scaffolds and the ordering of scaffolds within centimorgan positions could not be determined. In total, we have placed 807 Mb of genomic sequence onto chromosomal scaffolds, representing 58% of the base pairs in the current genome assembly and 60% of the annotated genes. We were also able to assign an additional 66 scaffolds to chromosomes but not to specific positions. Combined with the previous integrated map, the total number of scaffolds currently assigned to chromosomes is 691, representing 909 Mb and 66% of the genome.

### Synteny

**Figure 4:**
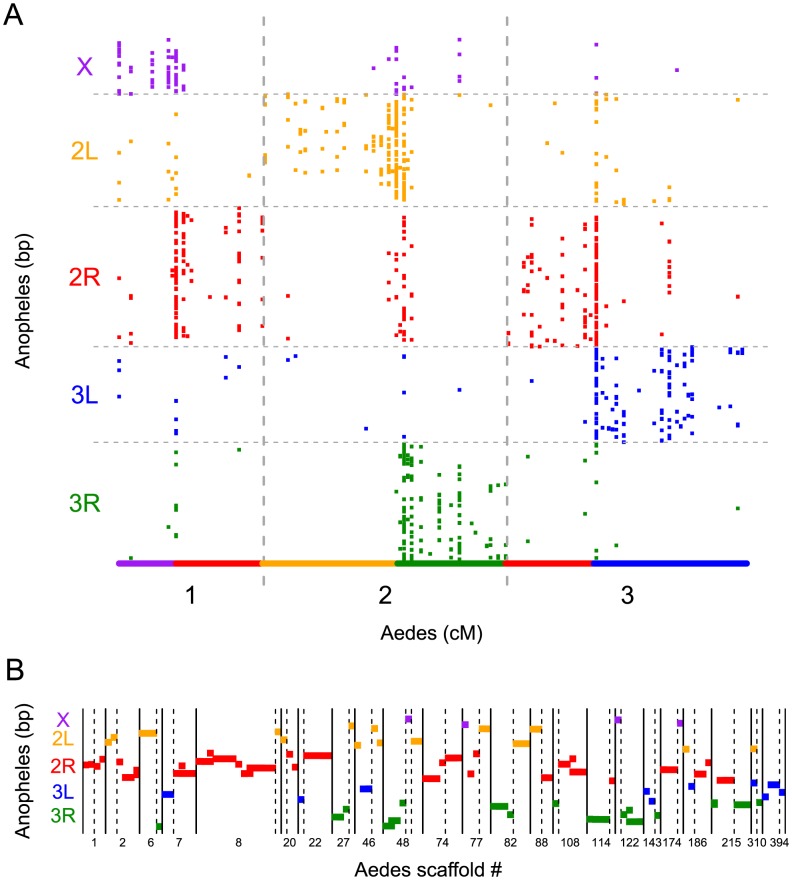
Synteny between the chromosomal arms of *Ae. aegypti* and *An. gambiae* can be used to corroborate scaffold misassemblies in *Ae. aegypti*. A) Synteny is maintained between chromosomal arms in *Ae. aegypti* and *An. gambiae*. Each square represents a 1∶1 orthologous gene (>70% identity) between species. The position is plotted as the centimorgan position on the genetic map for *Ae. aegypti* and the base pair position along the chromosomal arm in *An. gambiae*. The solid line at the bottom illustrates the syntenic relationships that have previously been observed [11]. B) Misassemblies are supported by breaks in synteny in cases where both halves of a misassembled scaffold have orthologous genes in *An. gambiae*. Each point along the x-axis represents a contig with an ortholog in *An. gambiae* and its location on the y-axis represents its position in *An. gambiae*. Dotted line represents scaffold misassembly breakpoints that were detected using the genetic map.

Synteny has previously been observed between whole chromosomal arms in *Ae. aegypti*, *An. gambiae*, and *C. pipiens* [11], [12]. We find support for the syntentic relationships between *Ae. aegypti and An. gambiae* that have previously been observed [11] (Figure 4A). Within chromosomal arm, we find no strong linear relationship between physical position on the *An. gambiae* chromosome and genetic position in *Ae. aegypti*, suggesting that fine-scale gene order has not been conserved over evolutionary time. Strikingly, we find that misassemblies that are found using the genetic map tightly correspond to breaks in synteny between the two species (Figures 1B, 4B). In 21 of 23 instances with orthologous gene pairs between *Ae. aegypti* and *An. gambiae*, misassemblies detected in the genetic map are accompanied by breaks in synteny (Figure 4B). In two instances (supercont1.1 and supercont1.394), misassemblies occur within chromosomal arms in one of the species and could not be supported by synteny. However, supercont1.1 has previously been demonstrated to be misassembled [20].

### Recombination rates

**Figure 5:**
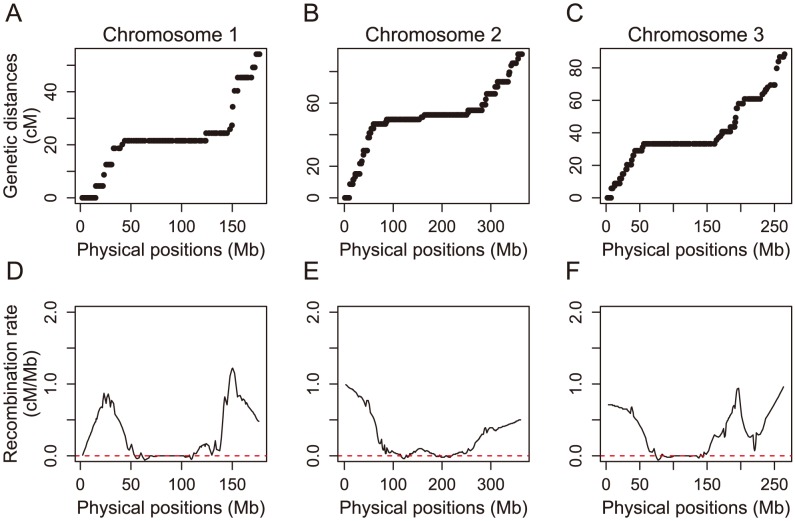
The relationship between genetic (cM) and physical map (Mb) positions and estimated local recombination rates across the three chromosomes of *Ae. aegypti*. The physical length was measured as the number of base pairs mapped to a particular genetic position for chromosomes A) 1, B) 2, and C) 3. Local recombination rates for chromosomes D) 1, E) 2, and F) 3 show depressed recombination in the centromeric regions of each chromosome.

Our high density genetic linkage map gives insights into rates of recombination across the *Ae. aegypti* genome. The rate of recombination per nucleotide can be obtained by comparing the genetic map (centimorgans) with the physical map of the genome (numbers of nucleotides). We estimated physical lengths by arranging the genome on the linkage map and summing the scaffold sizes — although we caution that this represents approximately 58% of the genome, so we are underestimating physical lengths (Figure 5A). Our genome wide average recombination rate and overall genetic map lengths are consistent with those found previously for *Ae. aegypti* [14] (Table 1), which are lower for those previously shown for both *Drosophila melanogaster* and *An. gambiae* [38]. Indeed, the recombination rate appears to be depressed across the entire length of the chromosome compared to what has been shown for the other two insects.

Estimated recombination rates vary across the chromosomal arms, with the lowest rates found near the centromeres in all three chromosomes (Figure 5B). This reduction in the recombination rate near the centromere of each chromosome is clearly seen on the genetic map, where we find 82–107 Mb regions — up to 47% of the chromosome — that map to the same or similar genetic positions (Figure 5A). This same pattern of reduced recombination rates near centromeres has been observed in the *D. melanogaster* and *An. gambiae* [39]–[41], but the physical distance that this spans (up to 20% of a chromosome in *D. melanogaster* and up to 40% in *An. gambiae*) is higher compared to both other insects.

Natural selection is ineffective in regions of the genome with low recombination rates, which can lead to these regions accumulating non-coding repetitive sequences, often derived from transposable elements [42], [43]. We therefore investigated whether the large genome size of *Ae. aegypti* could be explained by the preferential accumulation of non-coding sequence in regions of suppressed recombination. If this were the case, then we would have expected a higher gene density, a greater proportion of exonic sequence and shorter introns in higher recombination regions of the genome. Of the three, we only found a slight correlation between recombination rate and average intron length (Figure S3; Spearman correlation, gene density: ρ = −0.012, *P* = 0.784; proportion exonic sequence: ρ = −0.16, *P* = 0.781; average intron length: ρ = −0.094, *P* = 0.034). However, the average intron length in the low recombination region around centromeres is only slightly higher than the rest of the genome. Overall, the large genome size cannot be attributed to the accumulation of non-coding sequence in low recombination regions of the genome.

### Mapping genes affecting disease transmission

**Figure 6:**
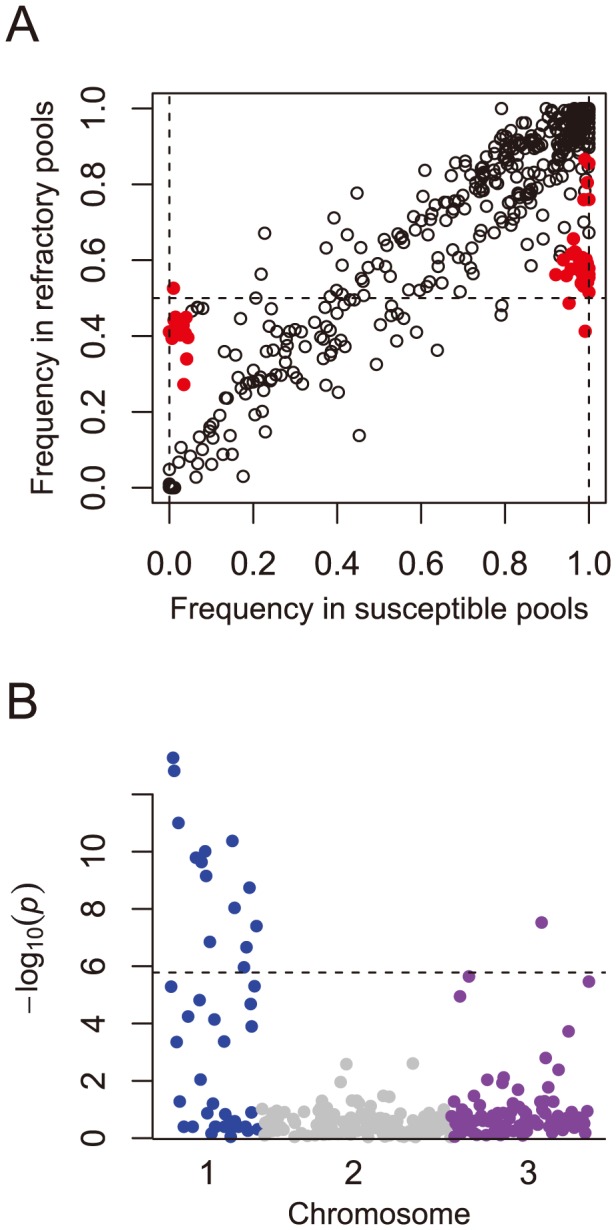
Resistance to *B. malayi* is determined by a single locus that is dominant in action and maps to the first chromosome. A) Average frequencies of the reference allele (the SNP allele in the published genome) in the pools of refractory versus susceptible mosquitoes. A cluster of markers with ∼100% frequency in susceptible pools (vertical dotted lines) and ∼50% frequency in refractory pools (horizontal dotted line) is consistent with resistance being determined by a single locus that is dominant in action. Red points indicate statistically significant differences in allele frequencies between refractory and susceptible pools at a genome-wide significance of *P*<0.01. B) Manhattan plot of the minimum *P*-values for each scaffold, with the x-axis representing a physical map created from the linkage map. Only markers from contigs used to assemble the linkage map are shown here (see Table S2). Dotted line indicates significance at a genome-wide significant of *P*<0.01.

Our assembly of the *Ae. aegypti* genome onto chromosomal scaffolds will facilitate the mapping of genes affecting disease transmission. To illustrate this and test the accuracy of our chromosomal scaffolds, we mapped resistance to *B. malayi*. We crossed a resistant and susceptible strain, and backcrossed the progeny to the susceptible parent. The progeny of this backcross were then fed on infected blood and dissected to examine whether the worm developed. In previous laboratory crosses, resistance has been nearly Mendelian in action and mediated by a sex-linked locus that is dominant in action [15], [17]. The segregation pattern we observe is consistent with this model, with significant markers having an average allele frequency difference of 38% between resistant and susceptible pools of mosquitoes (Figure 6A). We map resistance to *B. malayi* to the p arm of the first chromosome (Figure 6B), with the minimum *P*-value found at 0 cM and the maximum effect size at 12.5 cM. The peak could not be narrowed further with this approach due to the extremely low rate of recombination in *Ae. aegypti*. The maximum allele frequency difference observed in this region is 52% (Figure 6A), with the resistant individuals being heterozygous and susceptible individuals being homozygous for the putative susceptible allele (Figure 6A). A scan of the scaffolds in this region reveals a number of known immune response genes, including homologs of members of the Toll pathway, *Toll1A*, *Toll5A*, and *Spz4*, as well other genes that could potentially be related to resistance to *Brugia* (Table S4).

This analysis suggests that there are still misassemblies remaining in the genome. Figure 6 shows only markers which are on contigs that contained a marker on our genetic map (Table S2), so their chromosomal position is unambiguous and robust to errors in the scaffolding. However, if we include markers found anywhere on the scaffold, we find a scattering of associations across the genome (Figure S4). As the segregation pattern strongly suggests a single locus mediating resistance, and the significant markers on other chromosomes show the same segregation pattern (Figure 6A), this is likely because a number of scaffold misassemblies remain. Despite this evidence of remaining misassemblies, the high correlation with of our results with previous maps (Figure 3), the level of synteny between *Ae. aegypti* and *An. gambiae* (Figure 4), and the strong association signal on Chromosome 1, suggest that the majority of scaffolds are correctly placed, including many which have never before been associated with *Brugia* resistance.

### Conclusions

Genome-wide studies will be greatly facilitated by an improvement of the understanding of the organization of genome of *Ae. aegypti*. Recent progress has been made in advancing the physical map of the chromosomes, which currently represents approximately 13% of the genome. Here we present an improved genetic linkage map that places 58% of the genome onto chromosomal scaffolds. We have detected scaffold misassemblies in approximately 13% of the scaffolds with multiple markers, including in 6 of the 10 largest scaffolds in the genome. However, our data suggest that scaffold misassemblies remain to be detected, which needs to be addressed before truly complete chromosomal scaffolds are made. We confirm syntenic relationships between *Ae. aegypti* and *An. gambiae* chromosomal arms and demonstrate that this synteny can be useful for correcting the genome assembly of *Ae. aegypti*. Using our chromosomal scaffolds, we map *Ae. aegypti* resistance to the filarial nematode *B. malayi*, confirming its location on the p-arm of the first chromosome, and use our chromosomal scaffolds to generate a list of candidate genes underlying this trait. We also find that *Ae. aegypti* has low average recombination rates across all three chromosomes, with greatly reduced rates found in large regions flanking the centromeres.

## Supporting Information

Figure S1 **Larger scaffolds are more likely to have detected misassemblies independent of the number of markers.** For all scaffolds with more than a single marker, misassembled scaffolds have A) a higher average size but B) no significant difference in the number of markers. The probability of a scaffold with two or more markers having a misassembly detected was modeled using a general linear model with a binomial error, with the number of markers and contig length as explanatory variables. The scaffold size has a significant effect on the probability of detecting a misassembly significant effect on the probability of a misassembly (*z* = 6.4, *P*<10^−9^), but the number of markers did not (*z* = 0.8, *P* = 0.40).
(EPS)

Figure S2 **Larger scaffolds are more likely to have markers and to have detectable misassemblies.** Scaffolds were put into bins by decreasing length, and the percentages of scaffolds with markers (blue) and with misassemblies (red) were calculated for each bin. The cumulative percentage of the genome covered with the genetic map is shown in black. The percentage of scaffolds with misassemblies was measured as the number of misassembled scaffolds in each bin divided by the number of scaffolds in that bin with more than two markers (the minimum number required to detect a misassembly).
(EPS)

Figure S3 **Recombination rate is marginally correlated with average intron length but not with gene density or proportion exonic sequence per scaffold.** Recombination rate (cM/Mb) is not correlated with A) gene density or C) proportion exonic sequence and is slightly correlated with B) mean intronic sequence per gene (Spearman correlation, gene density: ρ = −0.012, *P* = 0.784; proportion exonic sequence: ρ = −0.16, *P* = 0.781; average intron length: ρ = −0.094, *P* = 0.034).β
(EPS)

Figure S4 **Mapping of resistance to** ***B. malayi*** **to chromosomal scaffolds.** Manhattan plot of the minimum *P*-values for each scaffold, with the x-axis representing a physical map created from the linkage map. The segregation pattern suggests a single locus (Figure 6A), however mapping of significant markers to all three chromosomes suggest that misassemblies remain. This pattern is removed by only plotting markers from contigs used to generate the linkage map (see Figure 6B).
(EPS)

Table S1 **Linkage groups were assigned to chromosomes based on chromosomal location in** [**20**] **.** Each cell value represents an individual scaffold. Chi-squared test, Χ^2^ = 95.6, df = 4, p<0.0001.
(XLSX)

Table S2 **Linkage map of** ***Ae. aegypti*** **with positional information.** Chromosome number, centimorgan position, misassembly indicator, old and new name, coordinates, length, and position within chromosome and genome are given for each scaffold (Excel sheet “genetic_map”). To facilitate plotting, positions of scaffolds that span neighboring centimorgan positions have been averaged so that each scaffold only appears once on the map. Individual contigs containing markers are listed with their position on the genetic map (Excel sheet “contigs”). Contig names refer to names in AGP file available at http://www.vectorbase.org. Markers with too much missing data to be included in the genetic map were used to assign additional scaffolds to chromosomes (Excel sheet “chromosome_only”).
(XLSX)

Table S3 **Misassembled scaffolds and their breakpoints.**
(XLSX)

Table S4 **Immunity genes that fall within the region with the maximum effect size and minimum** ***P*** **-value (0–12.537 cM) on chromosome 1.**
(XLSX)
